# Supplementary material for: HIVprotI: an integrated web based platform for prediction and design of HIV proteins inhibitors
Source: J Cheminform. 2018 Mar 9;10:12. doi: 10.1186/s13321-018-0266-y (PMC5845081; doi:10.1186/s13321-018-0266-y)
Supplement: Supplementary file 2 — Additional file 2. Source code of HIVProtI web server. [file 13321_2018_266_MOESM2_ESM.zip › HIVprotI_Source-code/hivprotI/weka-3-6-6/doc/weka/gui/beans/DataFormatListener.html]

DataFormatListener


---


|  |  |  |
| --- | --- | --- |
| **Overview** \| **Package** \| **Class** \| **Tree** \| **Deprecated** \| **Index** \| **Help** \| **Weka's home** | |  |
| **PREV CLASS**   **NEXT CLASS** | **FRAMES**    **NO FRAMES**     **All Classes** |  |
| SUMMARY: NESTED \| FIELD \| CONSTR \| METHOD | DETAIL: FIELD \| CONSTR \| METHOD |  |


---


## weka.gui.beans Interface DataFormatListener

**All Known Implementing Classes:**: ClassAssignerCustomizer

---

``` public interface DataFormatListener ```

Listener interface that customizer classes that are interested
in data format changes can implement.

**Version:**
:   $Revision: 1.3 $

**Author:**
:   Mark Hall

---

| **Method Summary** | |
| --- | --- |
| `void` | `newDataFormat(DataSetEvent e)`             Recieve a DataSetEvent that encapsulates a new data format. |

**Method Detail**

### newDataFormat

```
void newDataFormat(DataSetEvent e)
```

:   Recieve a DataSetEvent that encapsulates a new data format. The
    DataSetEvent may contain null for the encapsulated format. This indicates
    that there is no data format available (ie. user may have disconnected
    an input source of data in the KnowledgeFlow).

    :   **Parameters:**: `e` - a `DataSetEvent` value


---


|  |  |  |
| --- | --- | --- |
| **Overview** \| **Package** \| **Class** \| **Tree** \| **Deprecated** \| **Index** \| **Help** \| **Weka's home** | |  |
| **PREV CLASS**   **NEXT CLASS** | **FRAMES**    **NO FRAMES**     **All Classes** |  |
| SUMMARY: NESTED \| FIELD \| CONSTR \| METHOD | DETAIL: FIELD \| CONSTR \| METHOD |  |


---
